# Reengineering Tumor Microenvironment with Sequential Interleukin Delivery

**DOI:** 10.3390/bioengineering8070090

**Published:** 2021-06-30

**Authors:** Marxa L. Figueiredo, Rachel Letteri, Delphine Chan-Seng, Shreya Kumar, Cosette M. Rivera-Cruz, Todd S. Emrick

**Affiliations:** 1 Department of Basic Medical Sciences, Purdue University, 625 Harrison St., West Lafayette, IN 47907, USA; kumar308@purdue.edu (S.K.); riveracr@purdue.edu (C.M.R.-C.); 2 Purdue Center for Cancer Research and Purdue Institute for Drug Discovery, Purdue University, West Lafayette, IN 47907, USA; 3 Department of Chemical Engineering, University of Virginia, Charlottesville, VA 22904, USA; rl2qm@virginia.edu; 4 Université de Strasbourg, CNRS, Institut Charles Sadron UPR22, F-67000 Strasbourg, France; Delphine.Chan-Seng@ics-cnrs.unistra.fr; 5 Department of Polymer Science & Engineering, University of Massachusetts, 120 Governors Drive, Amherst, MA 01003, USA; tsemrick@mail.pse.umass.edu

**Keywords:** Interleukin-27, targeted IL-27pepL, Interleukin-18, prostate cancer, immune effector signatures, sonoporation, sequential delivery

## Abstract

Some cytokines can reengineer anti-tumor immunity to modify the tumor micro-environment. Interleukin-27 (IL-27) can partially reduce tumor growth in several animal models, including prostate cancer. We hypothesized that addition of IL-18, which can induce the proliferation of several immune effector cells through inducing IFNγ could synergize with IL-27 to enhance tumor growth control. We describe our findings on the effects of IL-27 gene delivery on prostate cancer cells and how sequential therapy with IL-18 enhanced the efficacy of IL-27. The combination of IL-27 followed by IL-18 (27→18) successfully reduced cancer cell viability, with significant effects in cell culture and in an immunocompetent mouse model. We also examined a novel chimeric cytokine, comprising an IL-27 targeted at the C-terminus with a short peptide, LSLITRL (27pepL). This novel cytokine targets a receptor upregulated in tumor cells (IL-6Rα) via the pepL ligand. Interestingly, when we compared the 27→18 combination with the single 27pepL therapy, we observed a similar efficacy for both. This efficacy was further enhanced when 27pepL was sequenced with IL-18 (27pepL→18). The observed reduction in tumor growth and significantly enriched canonical pathways and upstream regulators, as well as specific immune effector signatures (as determined by bioinformatics analyses in the tumor microenvironment) supported the therapeutic design, whereby IL-27 or 27pepL can be more effective when delivered with IL-18. This cytokine sequencing approach allows flexible incorporation of both gene delivery and recombinant cytokines as tools to augment IL-27’s bioactivity and reengineer efficacy against prostate tumors and may prove applicable in other therapeutic settings.

## 1. Introduction

Some cytokines can reengineer anti-tumor immunity to modify the tumor microenvironment. Interleukin (IL)-27 can serve as a therapeutic agent for malignant tumors based on its important role in immunomodulation [1] and its low toxicity profile [2]. IL-27, a member of the IL-12 cytokine family, is composed of subunits IL-27p28 and Epstein-Barr virus-induced gene 3 (*EBI3*), and signals through the IL27RA *(WSX1)* and IL6ST *(gp130)* receptor pair. IL-27 signaling induces T-box transcription factor 21 (Tbx21) and Interferon gamma (IFNγ), promoting initiation of T helper (Th)1 differentiation [3]. IL-27 also has direct transcriptional effects on several cell types, including tumor cells [4], and is able to induce natural killer (NK) and cytotoxic T lymphocyte responses, while reducing angiogenesis through CXCL9-10 upregulation [5]. 

In the present report, we have examined the impact of exogenous IL-27 as an immune-modulating therapy for prostate tumors in cell culture and in vivo. We administered IL-27, either as a recombinant cytokine in vitro or via skeletal muscle (plasmid DNA delivery through sonoporation or sonodelivery), an in vivo administration approach we have utilized in previous studies [6]. Although our previous work indicated that IL-27 could inhibit prostate tumor growth, one limitation was that tumors only showed ~50% growth reduction compared to controls [7]. We hypothesized that addition of IL-18, which can induce the proliferation of several immune effector cells through inducing IFNγ, major histocompatibility complex (*MHC*) class I expression, and also by inhibiting angiogenesis, could synergize with IL-27 to enhance tumor growth control. IL-18 belongs to the IL-1 family of cytokines [8] and has shown promise for inhibiting the growth of prostate adenocarcinoma by ~70% through immune potentiation (enhancing CD4, CD8, and NK cells) [9]. To the best of our knowledge, IL-27 and IL-18 had not yet been combined or sequenced in any in vivo tumor model prior to the present report. Limited in vitro data showed enhanced immune effector activity when NK cells were ‘primed’ with IL-27 [10], and had enhanced cytotoxic effect against tumor cells in culture, but the potential for in vivo tumor reduction remained untested. IL-27 was shown to enhance IFNγ production in a synergistic manner with IL-18 through enhancing Tbx21 expression but without upregulating IL4, IL10, IL17, IL6, or TNF [10]. Our approach of sequentially delivering cytokines can incorporate both gene delivery and recombinant cytokines as tools to help augment their bioactivity and antitumor efficacy. We tested single or sequential IL-27 and IL-18 therapies both in cells and in vivo, finding significant promise for the sequential 27→18 method for prostate tumors. 

In another recent report, we discovered the promise of a new IL-27 cytokine, targeted at the C-terminus with a short ‘peptide L’ (pepL, LSLITRL), which binds the interleukin 6 receptor α (IL-6Rα) upregulated in tumor cells [11] for reducing prostate tumor growth (IL27pepL or 27pepL) [4,12]. We thus compared the 27→18 cytokine sequence with 27pepL monotherapy and also with a 27pepL→18 sequence in this study. We utilized RNA sequencing (RNAseq) to examine pathways and potential modes of action in tumors, finding many common yet some unique pathways and immune effectors that 27pepL might utilize alone, or in combination with IL-18, to halt tumor growth. Overall, we found that the sequential approach (IL-27 or 27pepL-targeted) allows one to flexibly incorporate both gene delivery and recombinant cytokines as tools to augment a cytokine’s bioactivity and efficacy against prostate tumors.

## 2. Materials and Methods

### 2.1. Cell Culture and In Vitro Assays

Mouse TRAMP-C2-Ras cells (TC2R), are modified from the parental TRAMP-C2 prostate adenocarcinoma cells by lentiviral addition of an activated H-ras gene, and was developed and described in [13]. TC2R were maintained in DMEM:F12 (Mediatech, Manassas, VA, USA) with 10% FBS and 1× Antibiotic-Antimycotic (1xAA, Gibco). A Cell Counting Kit 8 (CCK8) (Dojindo) was used to assess the viability of tumor cells in 96-wells. Mouse cytokines were used at 10 ng/mL (IL-18; Medical & Biological Laboratories) and 50 ng/mL (IL-27; R&D Systems). For CCK8 assays, a baseline reading was performed according to the manufacturer’s specifications at day 1 post-seeding (3 × 10^3^ TC2R) in 96-wells, and then at days 3 or 6 with a media switch at day 3. For Luc assays, 10^4^ TC2R cells were seeded in white 96-wells (Corning), and transfection performed with Lipofectamine 2000 (Invitrogen) 24 h later with constructs, all of which contained the firefly luciferase (Luc) reporter gene, under the control of different regulatory elements, as follows: STAT1- and STAT3- (Panomics, Fremont, CA), IFNγ-, TNF-, IL4-, GATA2- (Addgene, Cambridge, MA), ARE(KLK3)-, TRAF6-, IL17α-, and NFATc1- (SwitchGear Genomics, Carlsbad, CA), NFKB- and AP1- (Clontech, Mountain View, CA), with 4% CMV-Bgal (Clontech) as a transfection control [14]. Prior to transfection, the media was changed to 0.5%FBS for 6–8 h, then the 1st cytokine was added with FBS to reach a 2% FBS final concentration. Twenty-four hours later, the second cytokine was added, and ~16 h later, media was aspirated and 50 mL 1xPassive Lysis Buffer (Promega, Madison, WI, USA) added to each well. Five microliters of lysate were used with a Luminescent Beta-galactosidase Detection Kit II (Takara Bio, Mountain View, CA, USA) and 45 uL used in a Luciferase Assay with a Glomax luminometer (Promega, Madison, WI, USA), with results expressed as RLU/sec (10 s integration).

### 2.2. Real Time PCR, Ingenuity Pathway Analyses (IPA), and CamcAPP

For qPCR, we treated TC2R cancer cells with mouse recombinant cytokines IL-27 and/or IL-18, and collected RNA at 24 h post-transfection (RNeasy kit, Qiagen). The cDNA synthesis and qPCR followed published procedures [4,15], with mouse-specific primers. Real time qPCR data were input into Ingenuity Pathway Analysis (IPA, Qiagen), as has been described [4]. For RNAseq data, putative regulator Gene Set Enrichment Analysis (GSEA) was done using Qiagen’s Ingenuity^®^ Pathway Analysis software (IPA version 01-09-02, Qiagen Redwood City, CA, USA, www.qiagen.com/ingenuity (accessed on 2 June 2021)) on genes that passed nominal *p* < 0.05 in comparison to pMCS control treated TC2R tumors vs. other vector treatments using the “Canonical Pathways”, “Upstream Regulators” analyses with qPCR or RNAseq data, and “Graphical Summaries” to integrate data. Upstream regulators with significantly predicted activation or inhibition state (z-score of >2.0 or <−2.0) that also met the Benjamini-Hochberg (B-H) multiple testing correction *p-*value of <0.05 were reported. The B-H corrected *p*-value enables control of the error rate in analysis results to focus in on the most significant biological functions associated with the genes of interest. We utilized CamcAPP (the Cambridge carcinoma of the prostate app) to validate differentially expressed genes (*p* < 0.05; up- or down-regulated) from each therapy relative to the human prostate cancer Cambridge dataset (https://bioinformatics.cruk.cam.ac.uk/apps/camcAPP/ (accessed on 22 May 2021)) [16]. The dataset provided information relevant to clinical covariates such as Gene Profile or Survival, using analysis of variance with z-score transformed data. 

### 2.3. RNAseq Analysis

Total RNA was isolated from tumors (n = 4–6/group) that had been preserved in RNAlater, and kept at −80 °C. Total RNA was isolated using buffer RLT containing 20 mL of 2 M DTT per mL of buffer, and homogenized with a PRO200 homogenizer (MidSci, Valley Park, MO, USA), in three brief pulses at a mid-power of 10–15 s each, keeping the tissue cold by submerging tubes in ice for 30–60 s in between pulses. The lysate was processed using Qiagen RNAeasy (Qiagen, Germantown, MD, USA). Poly(A) RNA sequencing library was prepared following Illumina’s TruSeq-stranded-mRNA protocol and conducted by LC Sciences (Houston, TX, USA). Poly(A) tail-containing mRNAs were purified using oligo-dT magnetic beads with two rounds of purification, and fragmented using divalent cation buffer at elevated temperature. Quality control analysis and quantification of the sequencing library were performed using an Agilent Technologies 2100 Bioanalyzer High Sensitivity DNA Chip. Paired-ended sequencing was performed on Illumina’s NovaSeq 6000. For transcript assembly, Cutadapt [17] and perl scripts in house were used to remove the reads that contained adaptor contamination and low quality bases. Sequence quality was verified using HISAT2 [18] and FastQC (http://www.bioinformatics.babraham.ac.uk/projects/fastqc/ (accessed on 1 August 2020)), and to map reads to the mouse genome, reads were assembled using StringTie [19], and transcriptomes merged using perl scripts and gffcompare. StringTie and edgeR [20] were used to estimate transcript expression levels. For differential expression analysis of mRNAs, StringTie was used by calculating fragments per kilobase million (FPKM). The differentially expressed mRNAs were selected with log2 (fold change) > 1 or log2 (fold change) < −1 and with statistical significance (*p* < 0.05) by edgeR. The datasets generated in this study have been submitted to Gene Expression Omnibus (GEO) in GSE178142.

### 2.4. Immune Cell Profiling Analyses Using RNAseq Data

For a general analysis of the immune landscape, we utilized XCell [21] and ImmuCellAI (Immune cell abundance identifier, ICAI) [22] to obtain microenvironment, immune, stromal, or infiltration scores. For a more detailed analysis of the immune cell profiling (>100 cell types), we utilized several recently characterized algorithms, including TIMER2.0 [23] (choosing PRAD-prostate adenocarcinoma), a comprehensive resource for analysis of immune cell infiltration among diverse cancers (http://timer.cistrome.org/ (accessed on 26 February 2021)). TIMER combines multiple algorithms [21,24,25,26,27,28], and we added analyses from the TIP [29] and ImmuCellAI [22] algorithms using TPM input, with prebuilt reference gene signatures. In addition, we utilized the Estimating the Proportion of Immune and Cancer cells (EPIC) algorithm (https://gfellerlab.shinyapps.io/EPIC_1-1/ (accessed on 26 February 2021)) [28] with bulk expression files using FPKM or counts input and reference expression files or signatures from gsea msigdb C7 (https://www.gsea-msigdb.org/gsea/msigdb/ (accessed on 26 February 2021)). (Supplementary Table S1) with https://biit.cs.ut.ee/gprofiler/convert (accessed on 26 February 2021) for formatting gene IDs. We normalized results within each algorithm and used clustvis [30] to represent data trends across algorithms.

### 2.5. In Vivo Studies

Animal care and all experimental protocols were approved by the Purdue University Animal Care and Use Committee (PACUC), and all experiments conform to all relevant regulatory standards (PACUC #1508001279). ARRIVE guidelines and recommendations from an NIH-sponsored workshop have been followed regarding experimental design and reporting standards. Tumor cells (TC2R [13], 5 × 10^5^) were implanted subcutaneously in C57BL6 males (8–10 weeks old) and sonoporation of 12.5 µg of pIL27ns (non-specific peptide at C-term), pIL27pepL (LSLITRL at C-term), or pMCS empty vector control (pcDNA3.1) with polymer rNLSd+ultrasound+MB was carried out (plasmids and sonoporation described in detail in [4]). We administered plasmids intramuscularly on day 4 (average tumor volume of ~30mm3), based on a report of sequential IL-12 and IL-27 therapy [5] and the recombinant IL-18 cytokine intratumorally on day 10, based on a prostate tumor model where localized IL-18 was promising therapeutically [9]. Mice (n = 4–6/group) were randomized by tumor size relative to treatment tested (pMCS, pIL27ns, plus or minus rIL-18). 

### 2.6. Statistical Analyses

Assays were performed in triplicate and values provided as mean ± SEM or 95% confidence interval. Comparisons were performed using unpaired t-tests for in vitro data or one-way analysis of variance analysis (ANOVA) for in vivo data (https://acetabulum.dk/anova.html (accessed on 2 February 2021)) using the Bonferroni t-test, and *p* < 0.05 was considered to indicate a significant difference.

## 3. Results

### 3.1. Sequential Administrion of IL-27 and IL-18 Impacted Prostate Tumor Cell Viability and Gene Expression

**Figure 1:**
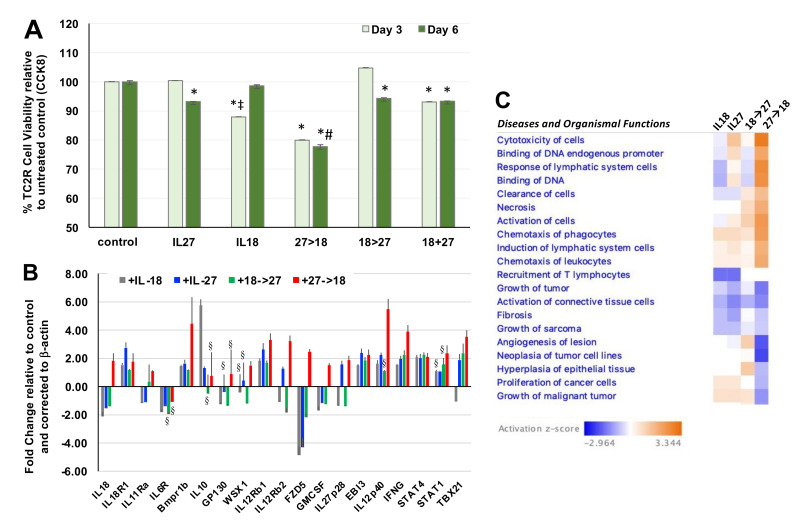
Impact of IL-27 and IL-18 administration sequence on prostate tumor cell viability. (**A**) The effect of treating prostate cancer cell line TC2R with cytokines was assessed at day 3 or 6, relative to day 1, using CCK8 cell viability assay with triplicate wells and expressed as a % relative to untreated control. Groups are untreated (control), IL-18, or IL-27 single cytokines, or combinations of cytokines. For the sequential administration, the baseline CCK8 is read at day 1, post seeding of cells. Then, on day 1, the first cytokine is given for 48 h, then media switched on day 3 to include both cytokines for another 48h, media switched again on day 5, and CCK read at day 6 (at 120 h). *, *p* < 0.05 relative to the ‘no treatment’ group. #, *p* < 0.05 relative to the IL-27 and IL-18 groups. ‡, *p* < 0.05 for the IL18 group relative to IL27; (**B**) Gene expression in TC2R cells (qPCR), mean value ± SD from triplicates; color bar, fold change over control untreated and corrected to β-actin housekeeping gene; All are significant (*p* < 0.05, two-tailed t-test) relative to control, except those marked with the symbol §, *p* > 0.05; (**C**) Diseases and Organismal Functions using Ingenuity Pathway Analyses. *Color bar*, activation z-score.

We examined the impact of using both cytokines first in recombinant form for initial activity screening. In this *in vitro* setting, we detected significant reduction in TC2R prostate cancer cell viability when IL-27 was used alone or co-administered with IL-18 relative to untreated control (Figure 1A; *, *p* < 0.05) and each single cytokine alone (#, *p* < 0.05). However, the only combination that reduced cell viability significantly relative to either single cytokine was the sequential administration of IL-27 and IL-18 (27→18). Next, we examined the effect of the 27→18 combination relative to single cytokines on differential gene expression in TC2R cells, selecting genes that our lab and others have shown to be modulated by IL-27 or IL-12. The 27→18 combination significantly upregulated key genes predicted to mediate a greater therapeutic impact such as IL12p40, Tbx21, STAT1, and IFNγ (Figure 1B, *, *p* < 0.05 compared to control). Interestingly, IL-18 can synergize with IL-12, and its IFNγ upregulation can act as a feedforward loop by inducing transcriptional activation at the IL-18 promoter [31]. Upregulation of STAT1/Tbx1 indicates induction of genes specific to the IL-27 pathway, whereas a cooperation between IL-27 and IL-18 may promote upregulation of IL12p40. In a reported IL-12→27 sequence, the growth of immunogenic colon and breast tumors was suppressed through a CD4 and CD8 T cell and IFNγ-based anti-tumor response [5]. This would suggest similar immune effector mechanisms for the 27→18 sequence in vivo. 

The qPCR results were input into the Ingenuity Pathway Analysis (IPA) software to examine diseases and organismal functions associated with these gene expression differences. IPA predicted general increases in cancer cell related functions of increased cytotoxicity of cells and reductions in growth of tumors with double cytokine treatments. Although limited to being a tumor cell culture, the gene expression patterns suggested that cytokines might modulate changes in the tumor cells, which could ultimately have an impact on immune cells when translated to an in vivo setting. Tumor microenvironment-related gene expression patterns suggested pathways relating to chemotaxis of phagocytes and recruitment of T lymphocytes (Figure 1C). Based on these functions, predicted to mediate the effects of the 27→18 sequence, we next sought to determine the impact of single or combination cytokines in vivo on the tumor-immune cell microenvironment with a broader study using RNA-seq.

### 3.2. The 27→18 Interleukin Sequence Enhanced Antitumor Activity In Vivo

In vivo, our design was modified due to previous success with delivery of IL-27 using gene therapy methods. We examined the effects of delivering plasmids, encoding either for an untargeted IL-27 (non-specific peptide at the C-term) or a IL27 targeting IL6Rα (IL27pepL), using intramuscular (I.M.) sonoporation relative to control vector (pMCS). The IL-18 was given as a recombinant cytokine (rIL-18) intratumorally, based on a previous study where it could yield partial antitumor responses when administered directly to prostate tumors [9]. 

**Figure 2:**
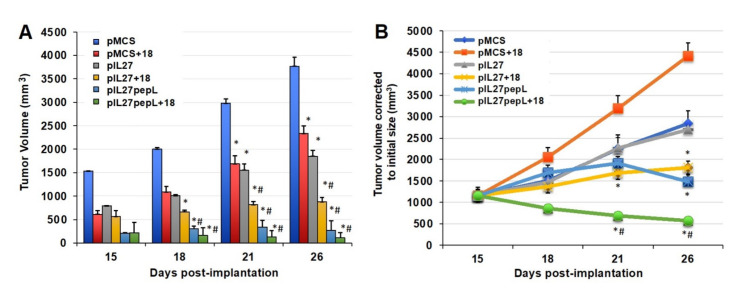
In vivo data shows tumor growth and gene expression impacted differently by the sequential 27→18 therapy. (**A**) Tumor volume (mm^3^) over time for the control (empty plasmid pMCS) and therapeutic groups (plasmids expressing untargeted IL27 (pIL27), targeted IL27 (pIL27pepL), or rIL18) for TC2Ras s.c. tumors (mean ± SEM); (**B**) Tumor volume (mm^3^) corrected to the initial tumor volume for examining the growth rate over time using ANOVA for comparing group differences (mean ± SEM). *, *p* < 0.05 compared to pMCS-treated control tumors; #, *p* < 0.05 compared to mice treated with single rIL18 or pIL27 (untargeted) therapies.

When testing IL-27 vectors and IL-18 together for potential synergy, we observed a significant reduction in tumor growth rate by day 15 relative to the control vector alone (Figure 2A). The sonodelivery of IL-27 has been previously described by our group [6,7] and utilized a polymer containing nuclear localization signals (rNLSd) complexed with plasmid DNA, delivered I.M. in the presence of microbubbles, and stimulated with ultrasound. By day 21, the 27→18 combination reduced tumor volume by ~21–52% relative to IL-27, IL-18, or vector control. By day 26, the 27→18 combination reduced tumor volume by ~76% relative to the control and by ~50–60% relative to single cytokine groups. Tumor growth rate was inhibited by ~40% for IL-18, ~49% for IL-27, and ~72% for 27→18-treated tumors, relative to control, between days 15 and 26. We next studied the impact of delivering a targeted form of the IL-27 vector, which we have recently described as more potent than wild-type untargeted IL-27 [4,12], because it has IL-27 functions enhanced by IL-6Rα inhibition. Interestingly, the IL-27 targeted to the IL6Rα (27pepL) was as effective as the dual therapy 27→18 (Figure 2B). Most notably, the addition of rIL18 to the 27pepL further magnified its effectiveness (Figure 2B), and produced an impressively efficacious therapeutic and the only group in which tumor volume was reduced over the initial tumor size.

We next examined the mechanisms of therapy on prostate tumors via RNAseq analyses, as described in Materials and Methods. We determined the number of significant (*p* < 0.05) differentially expressed genes (DEG) per treatment group. The IL-18 treatment group had 305 up- and 479 downregulated, IL-27 had 510 up- and 59 downregulated, and IL27pepL had 123 up- and 260 downregulated DEG. The 27→18 treatment group had 189 up- and 231 downregulated, and the 27pepL→18 had 166 up- and 253 downregulated DEG. From these totals, a list of the top 50 upregulated and bottom 50 downregulated DEG per treatment was input into camcAPP [32] for comparison with human prostate cancer datasets. The summary of correlations found with human prostate cancer DEG and with potential therapeutic impact are presented in Figure S1. Next, we will present the findings on therapy impact on global signaling pathways in prostate tumors by utilizing canonical pathway and upstream regulator analyses.

### 3.3. Overview of Canonical Pathways Modulated by Single and Double Therapies

**Figure 3:**
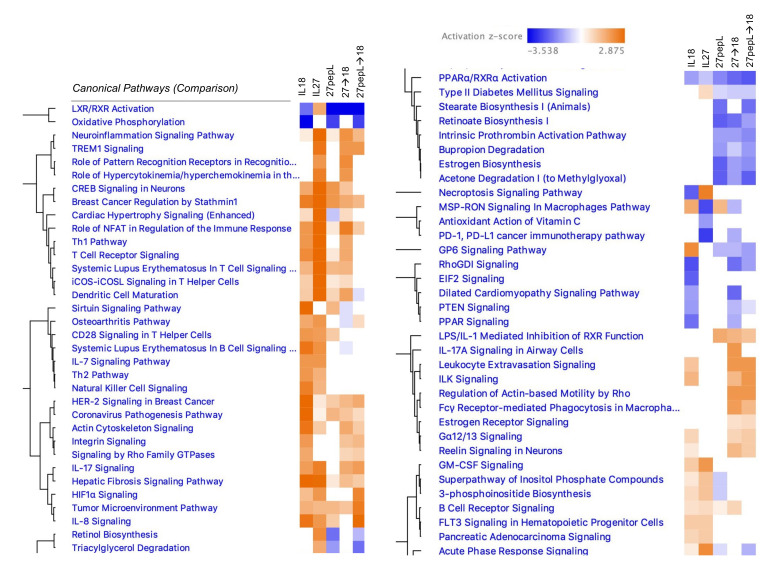
Canonical pathways comparison analysis across therapies (each lane represents a core analysis of a therapy group relative to control vector) for insights into general mechanisms of action. *Color bar,* activation z-score range, with *p* value < 0.05 as a cutoff. Hierarchical clustering was utilized in Ingenuity Pathway Analysis (IPA) to group pathways with similar patterns of z-score activation or inhibition across samples.

We examined the effect of therapies on tumors via the Ingenuity Pathway Analysis (IPA) software using the *canonical pathways* analysis, noting several trends among the therapy groups (Figure 3). For example, relative to control vector, most of the therapies (except for IL-27) showed a z-score predicting inhibition of the liver X receptor-retinoid X receptor (LXR-RXR) pathway. Additionally, there was a predicted inhibition in the oxidative phosphorylation pathway, except for those groups that contained IL-27. Common to the IL-18 and IL-27, single therapies were predicted activations of IL7, Th2, NK cell, GM-CSF, inositol phosphate-related pathways, and FLT3 signaling in hematopoietic progenitors. Common to the 27pepL groups were predicted reductions in retinol and triacylglycerol-related pathways. 

In IL-27-containing groups, several inflammation or infection-related pathways were predicted to be activated, including TREM1 signaling, pattern recognition receptors, neuroinflammation, and acute phase response, whereas inhibition of the PD1/PD-L1 cancer immunotherapy pathway was predicted. Dendritic cell maturation was predicted at higher activation scores in IL-27-containing groups, whereas CD28 signaling in Th cells was predicted as activated only for the single therapies. Several T-cell related pathways were predicted as activated in IL18- and/or IL-27-containing groups, including Th1 and TCR signaling. Common to the 27→18 and 27pepL→18 groups were activations of TREM1, IL17, Leukocyte extravasation, ILK, actin-based motility by Rho, Fγδ receptor-mediated phagocytosis in macrophages, and estrogen receptor signaling, among others. Finally, common to 27pepL and the double therapies, were a predicted increase in LPS/IL1-mediated inhibition of RXR function, and inhibitions of stearate- and retinoate-related biosynthesis pathways. 

### 3.4. Integrating Findings from the Canonical and Upstream Regulators Analyses for the Single Therapies 

We assessed the *upstream regulators* predicted in the different groups using the differentially expressed genes per each therapy (Figure S2). For the IL-18-treated tumors, the top 30 regulators included RICTOR, LARP1, TNF, PGR, TGFβ1, CSF1, SIRT3, CPT1B, IL6, and WNT3a. The bottom 18 regulators included MLXIPL, MYC, MYCN, DDX5, DAP3, Lh (complex), TWNK, ADAM12, MRPL12, and FASN (Figure S2A). 

For IL-27-treated tumors, among the top 30 activated regulators were IFNγ, IFNα/IFNαR, STAT1, IL33, CSF2, NFkB, IRF7, TGM2, IL5, and SMARCA4 (Figure S2B). Among the bottom 30 inhibited regulators were mir-21, Irgm1, SOCS1, INSIG1, TRIM24, HOXA10, ETV6-RUNX1, SIRT1, IL-10RA, and PNPT1. For the 27pepL treatment, among the top 26 activated regulators were TNF, CSF1, CD40, SIRT3, IFNα, Ig (complex), IL18, TNF, IL3, and mir-33 (Figure S2C). Among the bottom 30 inhibited regulators were HNF1A, HNF4A, SMARCB1, ADIPOQ, PPARγ, NR3C1, FASN, PEPB1, FST, and LONP1. 

**Figure 4:**
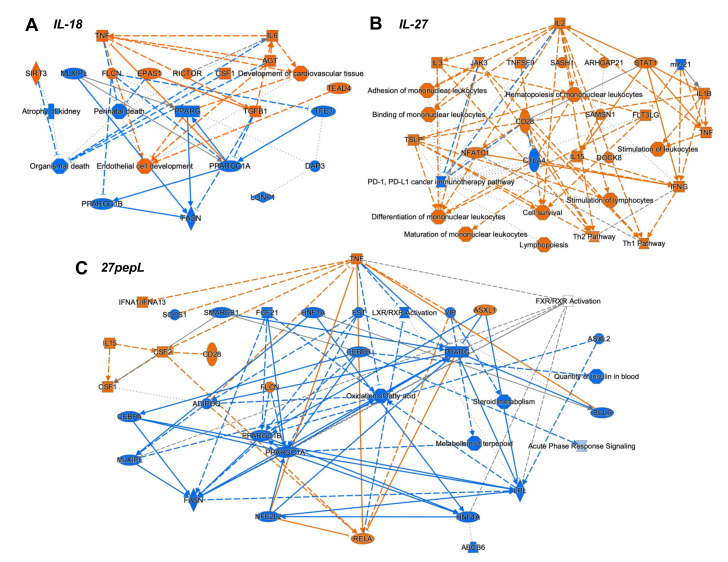
Graphical Summary for (**A**) IL-18, (**B**) IL-27, and (**C**) 27pepL single therapies.

To integrate the findings from the canonical pathways and upstream regulators analyses, we utilized the IPA graphical summary feature with a hierarchical representation of the key effects of each of the single therapies on prostate tumors (Figure 4). 

For IL-18, the major activated regulators were TNF and IL6, and the inhibited network centered on PPARγ. Other key regulators included TGFB1, SIRT3, CSF1, EPAS1, and RICTOR. Several of these changes appeared to reduce functions associated with cell or organismal death or enhance development of endothelial cells. 

For IL-27, the major regulator was IL2, with links to several immune-signaling molecules including TSLP, IL3, JAK3, STAT1, CD28, IL15, IFNγ, IL1β, and TNF. Mir-21 and CTLA4 were downregulated. Several pathways were connected to the key regulators, including hematopoiesis, differentiation and binding of mononuclear leukocytes, stimulation of lymphocytes, lymphopoiesis, and Th1 and Th2 pathway activation. 

For the 27pepL group, the major regulator was TNF, with connections to IFNα1/13, CSF2-CSF1, IL15, CD28, and RELA. Also observed were inhibition of FGF21-PPARγC, oxidation of fatty acid, metabolism of terpenoids and steroids, and LXR/RXR activity. Other inhibited regulators were ADIPOQ and HNF1A/4A. Additional inhibited processes included insulin-related pathways and the acute phase response (inflammatory/infection) signaling.

### 3.5. Integrating Findings from the Canonical and Upstream Regulators Analyses for the Combination Therapies 

**Figure 5:**
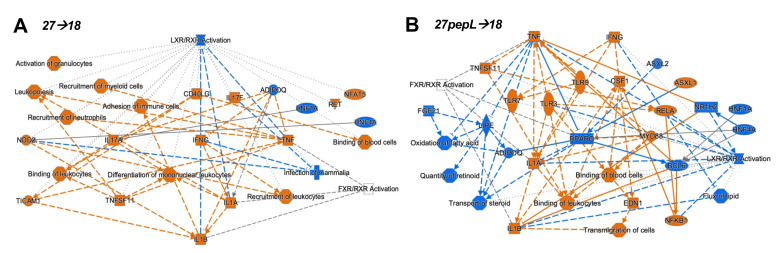
Graphical Summary for (**A**) 27→18 and (**B**) 27pepL→18 combination therapies.

For the combination therapies, we also assessed the *upstream regulators* predicted using the differentially expressed genes for each therapy. For the 27→18-treated tumors, the top 30 regulators included IFNγ, CSF1, IL21, LDL, MYD88, P38-MAPK, TLR3/4, CSF2, and IL2 (Figure S3a). Among the 30 bottom regulators were HNF1A/4A, ADIPOQ, IL10RA, and ACOX1, (Figure S3b). To integrate the findings from the canonical pathways and upstream regulators analyses, the IPA graphical summary feature was used to represent the key effects of each of the combination therapies on prostate tumors (Figure 5).

For the 27→18 therapeutic sequence, the major activated regulators were IFNγ, IL1α/β, and TNF, and the major inhibited pathway was LXR/RXR, with additional inhibited regulators including ADIPOQ and HNF1A/4A. Several activated regulators (IFNγ, NOD2) connected to the enhanced recruitment of leukocytes, leukopoiesis, differentiation of mononuclear leukocytes, whereas other regulators (TNF, TNFSF11) connected to the recruitment of myeloid cells. For 27pepL→18, the major activated regulators were IFNγ and TNF, whereas inhibited regulators included PPARγ, BCL6, FGF21, HNF1A/4A, NR1H2, and ADIPOQ, impacting functions such as transport/quantity of steroid, oxidation of fatty acids, or flux of lipid. Most of the activated regulators connected to activate processes such as binding of leukocytes or blood cells and transmigration of cells, with predicted activation of TLR3/7/9, IL1α/β, RELA, and NFkB1.

### 3.6. Immune Profiling Estimated Patterns for Several Effector Cells in Tumors Following Treatments

**Figure 6:**
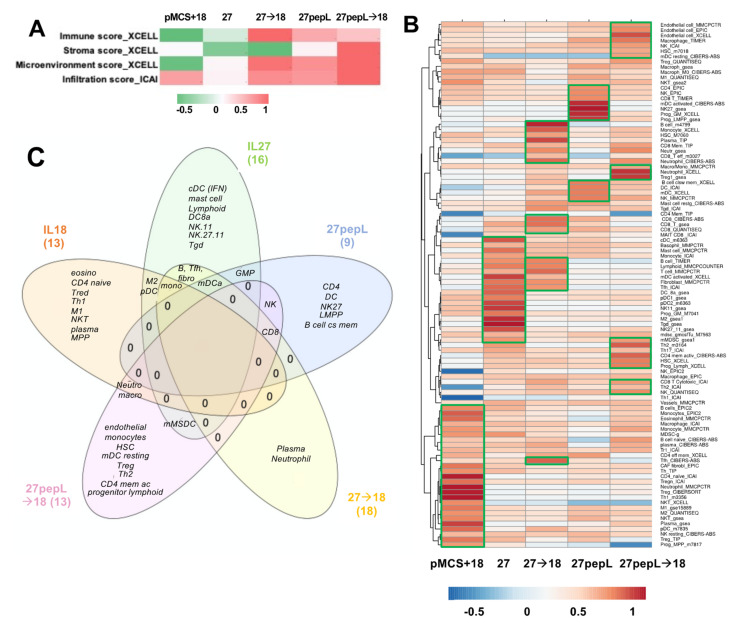
Tumor microenvironment and cellular profiling. (**A**) Immune, Stroma, Microenvironment, or Infiltration Scores. Color bar, range of relative values normalized to the highest score within each platform. (**B**) Immune cell profiling from utilizing bioinformatic tools with RNAseq tumor data, normalized within each platform, and clustered using Clustvis. Green boxes show the clusters enriched in each treatment group. (**C**) Venn diagram summarizing the main cell profile signatures detected in tumors with each therapy.

Based on the IPA analyses identifying many regulators and pathways relating to leucocyte recruitment and differentiation, we estimated that immune effectors would be differentially recruited to tumors and could underlie the therapeutic efficacy differences observed in this study. Utilizing bioinformatics tools for immune cell profiling with the RNAseq data, we performed an initial analysis focusing only on the broad infiltration or microenvironment scores using XCell and ImmucellAI, as described in Materials and Methods. The XCell analysis showed the highest *Immune Scores* (composite score of immune cell types) for 27→18, followed by 27pepL and 27pepL→18 (Figure 6A). 

The Stroma Score was the highest for 27pepL→18. The XCell Microenvironment Score (composite scores of immune cell types and stromal cell types) followed a similar trend as the ICAI infiltration score, with the highest being 27pepL→18, followed by lower scores for 27→18 and 27pepL. The only positive score for the IL18-treated tumors was the ICAI Infiltration Score, which estimates degree of tumor infiltration by immune cells.

A more detailed analysis was carried out next using various algorithms, as described in more detail in Materials and Methods. Clustering analysis of the results showed a cell type enrichment signature in the IL-18 treated tumors for several immune cell types consistent with the IPA and Reactome pathways implicated in therapy effectiveness, including monocytes, eosinophils, CD4 naïve, regulatory T (Treg), neutrophil, T helper 1 (Th1), macrophages including M1- or M2-polarized, natural killer T cells (NKT), plasma cells, plasmacytoid dendritic cells (pDC), and multipotent progenitor (MPP). For the IL-27-treated tumors, we detected signature enrichment for several cell types, including conventional DC (cDC), mast cells, monocytes, B cells, Lymphoid, myeloid DC (mDC) activated, fibroblasts, follicular T helper (Tfh), CD8a DC (DC8a), pDC, NK1.1 natural killer cells CD11b (NK11) and CD27 CD11b (NK27.11), granulocyte-monocyte progenitor (GMP), M2 macrophages, gamma delta T (Tγδ), and monocytic myeloid-derived suppressive cells (mMDSC) (Figure 6B). 

For the 27→18-treated tumors, we detected a cell type enrichment signature of B cells, monocytes, plasma cells, neutrophils, CD8 T effectors, and Tfh. For the 27pepL-treated tumors, we detected enrichment signatures of CD4 T, progenitors GMP and LMPP, B cell class-switching memory cells, three DC signatures including mDC activated, two CD8 T signatures, and three NK signatures including NK.27. For the 27pepL→18 treated tumors, we detected enrichment in endothelial, HSC, mDC resting, neutrophil, Treg, mMDSC, Th2, CD4 memory activated, progenitor lymphoid, CD8, two macrophage or macrophage/monocyte, two HSC, and two NK signatures.

Thus, overall, the therapies impacted multiple mechanisms relating to antitumor immunity; however, several pathways and immune cells might hinder therapeutic effectiveness, thus opening up avenues for future interventions that could be paired with the cytokine sequences presented here for achieving therapeutic synergy.

## 4. Discussion

In this manuscript, we describe the therapeutic administration of IL-27 in a sequential manner with IL-18 for treating prostate cancer. IL-27 has shown promise in halting tumor growth and mediating tumor regression in several cancer models, including prostate cancer. Previous work by Ziblat et al. [10] showed that IL-27 delivery could prime NK to IL-18’s effects of high-level IFN-γ and cytotoxic action towards Raji, T47D, and HCT116 tumor cells. We therefore hypothesized that the efficacy of our IL-27 gene delivery protocol for prostate tumors could be augmented if combined with IL-18 administration, especially if administered sequentially. For delivery of IL-27, we used intramuscular (I.M.) sonoporation *in vivo* (sonodelivery) of plasmids encoding either untargeted IL-27 (27) or targeted to the IL6Rα (IL27pepL or 27pepL), relative to control vector (pMCS) and in the absence or presence of rIL-18 (IL-18 or 18) intratumoral administration. We chose sonodelivery because we have recently utilized this strategy to achieve partial antitumoral responses for IL-27 [1,7], whereas intratumoral rIL-18 has yielded partial antitumor responses when administered directly to prostate tumors [9]. Initial studies *in vitro* used a qPCR screen with IPA analysis, which presents several limitations, including few directional effects (e.g., downstream functions and upstream regulators). However, this initial data served to choose the initial sequencing of 27→18 as the most promising for *in vivo* validation, as well as validating the priming concept from Ziblat et al [10], but for the first time in prostate tumor cells. Indeed, we observed a significant reduction in tumor growth rate, with the 27→18 combination most substantially reducing tumor volume relative to single cytokine groups. We also explored the impact of the targeted form of IL-27, which we have recently described as more potent than wild-type or untargeted IL-27 [4,12]. Supporting the recent data, the 27pepL was as effective as 27→18. Adding IL18 to the 27pepL (27pepL→18) further magnified its effectiveness, and in this treatment group the average tumor volume was stably controlled.

We next examined the mechanisms of therapy on tumors via RNAseq analyses and determined a comparison canonical pathways analysis across all therapies. Common to most therapies was a downregulation in the LXR/RXR pathway, except for IL-27 alone. The liver X receptors (LXR) are nuclear receptors that integrate metabolic (transcriptional control of lipid metabolism) and inflammatory responses in tumor and immune cells [33]. Their downregulation points to a residual inflammatory tumor microenvironment, which could be therapeutically manipulated to aid in rebalancing the tumor with activated M1 macrophages, for example. In IL-27-containing groups, although the LXR/RXR pathway was upregulated, several other inflammation or infection-related pathways were predicted to be activated, which could impact treatment positively or negatively depending on the cell types present. For example, the TREM1 pathway could be beneficially associated with M1 tumoricidal macrophages, and the predicted inhibition of the PD1/PD-L1 cancer immunotherapy pathway in IL27-containing groups could act to enhance T cell activation, proliferation, and survival [34]. 

Common to IL-18 and IL-27 single therapies were predicted activations of IL7, Th2, NK cell, GM-CSF, inositol phosphate-related pathways, and FLT3 signaling in hematopoietic progenitors. These suggest a potential impact on hematopoietic cell recruitment of both lymphocytic and mononuclear cells, aligning with the immune cell signature analyses. Common to the 27pepL groups were predicted reductions in retinol and triacylglycerol-related pathways, and these could potentially promote or reduce tumor cell growth (via Treg inhibition), depending on the context [35]. Anti-tumor T-cell related pathways were predicted as activated in IL-18- and/or IL-27-containing groups, including Th1 and TCR signaling. Dendritic cell maturation activation was predicted at higher levels in IL27-containing groups. Detrimental (Th2-promoting) pathways included CD28 signaling for both single therapies, and activation of TSLP, a hematopoietic cytokine, for the IL27 therapy group. Common to the 27→18 and 27pepL→18 groups were pathways of enhanced macrophage activity and leukocyte infiltration, and these may be pro- or anti-tumorigenic, depending on the context. IL1 and other proinflammatory mediators identified as activated in the 27pepL, and the double therapies could potentially be detrimental for controlling tumor growth. 

The integration of the canonical pathways and upstream regulators datasets led to graphical summaries that aided in interpretation of therapeutic effects. For IL-18, the major activated regulators were TNF and IL6, with key contributions from TGFB1 and CSF1, and an inhibited PPARγ network. These changes appeared to reduce functions associated with cell or organismal death or enhance development of endothelial cells, which could be detrimental for treating tumors. For IL-27, the major regulator was IL2, with links to many other immune related molecules. Upregulation of JAK-STAT molecules and IFNγ likely underlie the antitumor effect of this therapy and correlate with immune cell signatures detected in tumors. A pro-inflammatory environment (IL1β and TNF) could stimulate tumor growth but also promote tumoricidal M1 macrophage activity. Predicted upregulation of JAK3 and CD28 could inhibit the PD1/PDL1 pathway via CTLA4 inhibition, whereas miR-21 inhibition augments FLT3LG activation. FLT3LG promotes hematopoiesis of lymphocytes and mononuclear cells, and this is enhanced by TNF activation. NFATC1, TSLP, and IFNγ also connected towards predicted enhancement of mononuclear cell differentiation, stimulation of lymphocytes, lymphopoiesis, and Th1 and Th2 pathways. For 27pepL, the major regulator was TNF, with other effectors such as activated IFNα1/13 and CSF1/2, which can help enhance the antitumor effects of IL-27 in the tumor microenvironment, either by impacting lymphocytes (when connecting to IL15-CD28) or myeloid cells (when connecting to RELA activation). Inhibition of FGF21-PPARγ was central to a network of reduced fatty acid oxidation, metabolism of steroids, and LXR/RXR regulation. Interestingly, PPARγ plays a role in controlling inflammation, and its expression increases in advanced prostate cancer; thus, its inhibition has been proposed for prostate cancer prevention and treatment [36]. Other alterations with a tumor-inhibitory impact would be inhibition of ADIPOQ, associated with prostate cancer progression risk [37], and HNF1A/4A, recently proposed as oncogenic in pancreatic cancer [38]. Also inhibited were CEBPB and insulin-related processes, likely contributing to the reduced acute phase response (inflammatory/infection) signaling. 

For the 27→18 sequential delivery technique, the major activated regulators were IFNγ, IL1α/β, and TNF, all predicted to inhibit the LXR-RXR pathway. Another central inhibited regulator was ADIPOQ, and this was predicted to underlie the increases in TNF, IL17A, and IFNγ. Several regulators (e.g., IFNγ, NOD2) connected to enhanced recruitment of leukocytes, leukopoiesis, or differentiation of mononuclear leukocytes. TNF and TNFSF11 activation connected to the recruitment of myeloid cells, potentially relating to the detection of mDC cell signatures by the immune cell profiling analyses. For 27pepL→18, the major activated regulators were IFNγ and TNF, both impacting several molecules (HNF1A/4A, PPARγ, BCL6, FGF21, and NR1H2), which inhibited lipid or steroid transport/quantity, oxidation of fatty acids, and flux of lipid, functions which were connected through a network of reduced LXR-RXR activity. Most of the regulators activated processes such as the binding of leukocytes or blood cells, and transmigration of cells. Also connected through TNF-TNFSF11 were activated TLR3 and RELA, culminating in IL1α/β, NFkB1, and CSF1 activation. This network indicates a potential hub of myeloid cell signaling that could be manipulated therapeutically. 

Based on the many immune-related functions and regulating molecules by IPA analysis, we hypothesized that immune effectors would be differentially recruited to tumors and could underlie the therapeutic efficacy differences. Utilizing bioinformatics strategies for immune cell profiling with the RNAseq data, we observed high immune scores for the dual therapies and targeted IL27 that usually correlate with increases in effectors in tumors. Several immune signature scoring algorithms were used, and these methods have been found to yield results consistent with those derived from immunohistochemistry and related methods examining lymphocyte-specific expression patterns within cancer vs. non-cancer cellular compartments [23,28]. The multiple algorithms included TIMER2.0 (containing XCell, MCP-Counter, QuantiSeq, and others), EPIC, ICAI, and TIP, as described in Materials and Methods. These algorithms have been systematically benchmarked on a variety of tumor types along with characterization of gene signatures of sorted immune cell populations as controls [23,24]. However, the limitations are that these remain bulk tumor transcriptome profiling methods, utilizing gene signature enrichment analyses or deconvolution methods. With the increasing accessibility of single-cell technologies, there will be likely continued improvements in defining immune cell signatures and in computational estimation methods for tumor profiling. 

Clustering analysis of the results showed an enrichment in the *IL-18 treated* tumors for monocytes, macrophages including M1, M2, eosinophils, neutrophil, CD4 naïve, Treg, Th1, NKT, plasma, pDC, and progenitor MPP, some of which align with the functions of phagocytosis and leucocyte migration detected by other analyses. The effector cells Th1 and NKT would likely underlie some of the therapeutic efficacy seen in IL-18 treated tumors; however, the other immune-suppressive cell signatures detected (Treg, M2, pDC, macrophages) may hinder therapeutic efficacy. The detection of MPP progenitors, which very recent data links to later stages of tumor progression, could implicate a subpopulation able to generate pro- or anti-tumorigenic macrophages depending on the context [39]. Inflammatory signals in tumors might promote progenitor accumulation [40]. 

For the *IL-27-treated* tumors, we detected enrichment of signatures for DC (conventional or cDC (IFN-producing), myeloid DC or mDC (activated), DC8a (CD8a), and plasmacytoid DC or pDC), mast cell, monocyte, B cell, Lymphoid, fibroblast, Tfh, NK.11 (CD11b) and NK.27.11 (CD27/CD11b), granulocyte-monocyte progenitor (GMP), M2 macrophages, Tγδ, and monocytic MDSC (mMDSC). The effector cells (NK, Th1, other T cells, c/mDC) likely underlie the therapeutic efficacy observed in IL-27-treated tumors; however, there were other residual immune-suppressive cells (M2, mMDSC, pDC), which could hinder therapeutic efficacy. Tfh is a CD4 T helper subset (T follicular helper) was recently found to correlate with improved survival when increased in tumors and, along with B cells, can mediate the response to checkpoint inhibitors in mouse models of augmented mutation burden in breast cancer [41]. The GMP can partially underlie the increases in monocytic cells, although these also are able to induce Treg, and are often biased toward myeloid differentiation in tumors, worsening prognosis [42]. In future iterations of IL27 therapies, targeting the GMP stage for depletion may enhance efficacy by controlling myeloid cell burden [43]. The NK signatures detected have important implications, with NK.27 and NK.27.11 being more cytotoxic than NK.11 [44].

For the *27pepL-treated* tumors, we detected enrichment of signatures for CD4, NK/NK.27, CD8 T, mDC activated/DC, GMP and lymphoid-myeloid progenitors (LMPP), and B cell class-switching memory. These e-signatures were different than for untargeted IL-27, suggesting that the higher therapeutic efficacy of 27pepL might relate to certain combinations of effectors, such as NK.27 combined with CD8T, which can act in synergy [23], activated DCs, and/or progenitors including the LMPP, which may act as pro- or anti-tumorigenic, providing both lymphoid (antitumor) and myeloid (antitumor M1 or immune suppressive M2, MDSC) cells.

For the dual therapies, we detected, for *27→18-treated* tumors, enrichment of B cell, monocyte, plasma cell, Neutrophil, Tfh, CD8T effector/CD8, mDC activated, fibroblast, and Tfh signatures. CD8 signatures were more prominent for this treatment, and CD8 infiltrations correlate with better prognosis and are an important predictor of response to immune therapy [45]. For the *27pepL→18 treated* tumors, we detected enrichment of endothelial cells, macrophage/monocytes, NK, HSC, mDC resting, neutrophil, Treg, mMDSC, Th2, CD4 memory activated, progenitor Lymphoid, and CD8. These results suggest that several effectors could underlie the highest efficacy for this therapy combination, including NK, mDC, and CD8, although several immune suppressive cells may be residual following therapy, including Treg and mMDSC, as well as endothelial cells, which could be detrimental to therapy. Interestingly, HSC and progenitor cells retain a certain degree of mobility, with a small fraction of progenitors constantly recirculating between BM and peripheral blood, surveilling extramedullary sites and participating in local immune responses [46]. They are recruited to sites of injury [47] and react to inflammatory stimuli with proliferation differentiation into myeloid cells, as well as with secretion of chemokines and cytokines.

In conclusion, although some inflammatory regulators remain in the 27pepL and combination therapies, the signaling context of reduced PPARγ and LXR-RXR activation and the presence of certain immune cell effectors may help explain the higher therapeutic success of these modalities relative to the untargeted cytokine controls. There seems also to be a role suggested by detection of a CD8 T signature in the most effective therapies. Overall, the analyses utilized and their integration gave different insights into the therapies, suggesting some future directions to enhance the present IL27-based therapeutics, including the combination of 27pepL with inhibition of PD1/PD-L1, CSF1/2, or LXR-RXR ligands, which may target tumor and immune cells such as macrophages. Other potentially useful parameters to promote the efficacy of 27pepL-based therapeutics may involve inhibition of TSLP, TNF, and IL1α/β signaling that direct the immune balance towards CD8 T lymphocytes in tumors.

Furthermore, the present work may impact treatment of other disease types and cancer types, but with careful consideration of the application and the inflammatory status of the tissue(s) to be treated. For example, because IL-18 has recently been shown to have an unfavorable predictive role in some inflammatory contexts (non-alcoholic fatty liver disease (NAFLD) [48]), for certain therapeutic settings it might be best to utilize 27 or 27pepL single therapies. IL-27 has been reported to augment the anti-tumor effects of sorafenib on bladder cancer cells [49]; thus, there could be promise for utilizing 27-related therapies for this cancer type. One interesting approach could be to engineer IL-27 with a C-terminal peptide mimic of sorafenib as a novel therapy. Future preclinical studies might examine the potential for evolving IL-27-based therapies and combinations with other cytokines for the treatment of bladder cancer.

## Data Availability

Data is in process of deposit into GEO datasets, accession number pending.

## Supplementary Materials

The following are available online at https://www.mdpi.com/article/10.3390/bioengineering8070090/s1, Figure S1: Patterns of Gene Expression and their correlation with potential therapeutic benefit in human prostate tumors, Figure S2: Upstream Regulators (UR) from IPA analyses, single therapies, Figure S3: Upstream Regulators from IPA analyses for the combination therapies, and Table S1: Gene expression signatures utilized in the EPIC RNAseq cell profiling analyses.
