# Supplementary material for: Reengineering Tumor Microenvironment with Sequential Interleukin Delivery
Source: Bioengineering (Basel). 2021 Jun 30;8(7):90. doi: 10.3390/bioengineering8070090 (PMC8301035; doi:10.3390/bioengineering8070090)
Supplement: Supplementary file 1 [file bioengineering-08-00090-s001.zip › bioengineering-1269201-supplementary.pdf]

Supplemental Figures

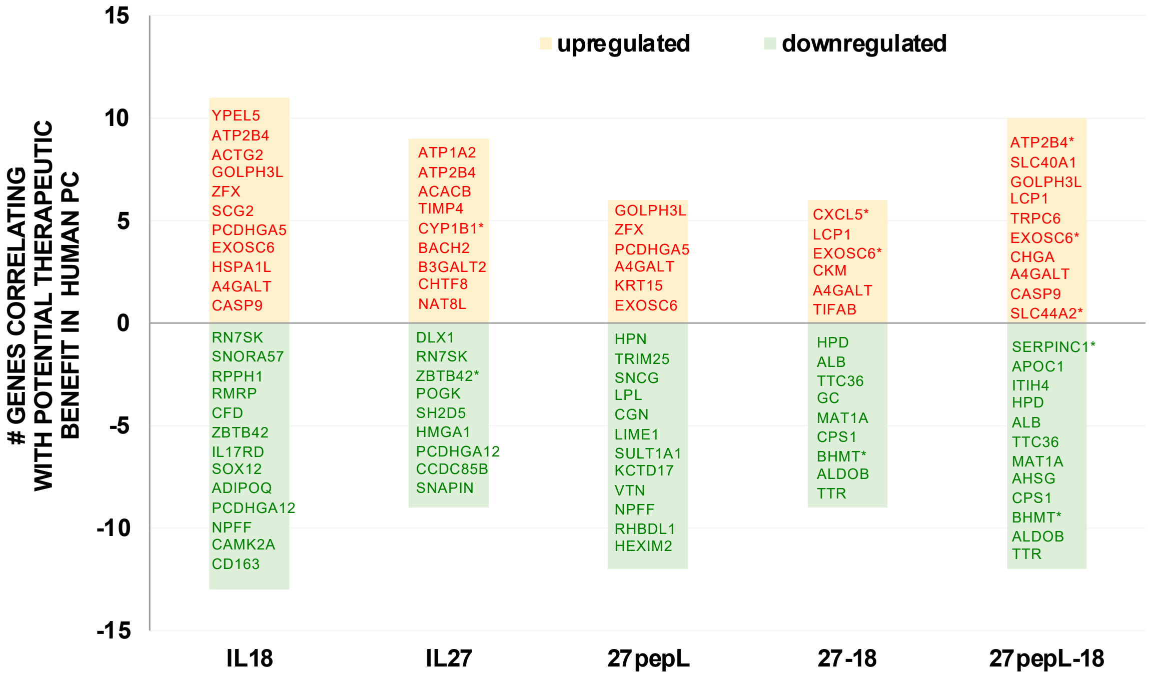

Figure S1. Patterns of Gene Expression and their correlation with potential therapeutic benefit in human prostate tumors. The Cambridge dataset was utilized with camcAPP and ANOVA analysis used to determine Gene Profile and Survival correlations at p<0.05. The top 50 or bottom 50 differentially expressed genes were used from each treatment for this comparison. Red, denotes upregulated genes and Green, denotes downregulated genes. *, gene expression pattern correlates with higher survival (using camCAPP recursive partitioning analysis at p<0.05).

A **IL18**

UR: top 30

18 UR	Molecule Type	Activation z-score	B-H corrected p-value
RICTOR	other	4.849	1.03E-09
LARP1	translation regulator	4.58	5.99E-13
TNF	cytokine	3.906	7.80E-06
PGR	ligand-dependent nuclear receptor	3.513	2.43E-02
TGFB1	growth factor	3.422	2.80E-07
CSF1	cytokine	3.406	1.32E-03
SIRT3	enzyme	3.301	1.97E-05
CPT1B	enzyme	3.19	7.84E-06
IL6	cytokine	3.115	2.31E-02
WNT3A	cytokine	2.965	1.22E-02
AGT	growth factor	2.946	8.25E-07
FLCN	other	2.798	1.04E-04
KLF4	transcription regulator	2.485	3.59E-02
SP1	transcription regulator	2.476	2.96E-02
KRAS	enzyme	2.454	3.79E-05
miR-2392	mature microRNA	2.441	8.25E-07
mir-2392	microRNA	2.439	8.25E-07
PDGF BB	complex	2.43	1.37E-03
ERBB2	kinase	2.429	1.12E-04
ETS1	transcription regulator	2.421	2.55E-02
NR1H4	ligand-dependent nuclear receptor	2.417	2.55E-02
NFAT5	transcription regulator	2.393	4.94E-02
PI3K (complex)	complex	2.386	2.20E-02
TEAD4	transcription regulator	2.373	1.43E-03
IL3	cytokine	2.297	4.27E-03
RET	kinase	2.217	8.84E-03
Ccl2	cytokine	2.177	4.34E-02
CTNNB1	transcription regulator	2.174	9.54E-16
PTH	other	2.158	3.93E-03
P38 MAPK	group	2.128	2.49E-03

UR: bot 18

PPARGC1B	transcription regulator	-2.02	1.07E-02
MALSU1	other	-2.236	6.24E-06
MRPL14	other	-2.236	1.84E-05
PTCD1	other	-2.236	1.48E-04
LONP1	peptidase	-2.309	8.06E-07
ALKBH1	enzyme	-2.449	8.25E-07
NSUN3	enzyme	-2.449	8.25E-07
CAB39L	kinase	-2.478	2.52E-05
FASN	enzyme	-2.607	2.56E-02
MRPL12	other	-2.613	1.16E-08
ADAM12	peptidase	-2.8	1.27E-02
TWNK	enzyme	-2.813	5.56E-06
Lh	complex	-3.139	3.77E-08
DAP3	other	-3.162	1.36E-11
DDX5	enzyme	-3.512	9.50E-09
MYCN	transcription regulator	-3.52	2.75E-08
MYC	transcription regulator	-4.096	8.46E-13
MLXIPL	transcription regulator	-5.251	4.32E-18

B **IL27**

UR: top 30

27 Upstream Regulator	Molecule Type	Activation z-score	B-H corrected p-value
IFNG	cytokine	8.313	2.13E-73
Interferon alpha	group	6.512	4.77E-37
STAT1	transcription regulator	6.454	4.43E-50
IL33	cytokine	6.319	9.45E-36
CSF2	cytokine	6.106	1.75E-28
NFkB (complex)	complex	5.901	1.62E-15
IRF7	transcription regulator	5.687	1.28E-22
TGM2	enzyme	5.599	3.93E-14
IL5	cytokine	5.552	3.23E-14
SMARCA4	transcription regulator	5.344	9.25E-09
Ifnar	group	5.222	7.20E-26
AGT	growth factor	5.213	4.99E-08
IRF3	transcription regulator	5.184	6.17E-19
SPI1	transcription regulator	5.158	2.56E-19
IL2	cytokine	5.144	4.33E-26
IFNA2	cytokine	5.11	1.44E-20
P38 MAPK	group	5.01	3.83E-09
IL1B	cytokine	4.821	7.81E-34
TLR3	transmembrane receptor	4.777	9.84E-12
TNF	cytokine	4.754	2.74E-46
NONO	transcription regulator	4.748	2.86E-12
Ige	complex	4.71	6.51E-20
ELAVL1	other	4.629	2.89E-11
TLR9	transmembrane receptor	4.585	9.84E-12
G protein alphai	group	4.583	3.36E-20
ARNT2	transcription regulator	4.583	4.81E-07
SIM1	transcription regulator	4.583	9.21E-07
TLR7	transmembrane receptor	4.571	3.07E-12
MYD88	other	4.567	2.02E-12
TLR4	transmembrane receptor	4.499	2.17E-19
[illegible]	[illegible]	[illegible]	[illegible]

UR: bot 30

COP1	enzyme	-2.97	3.82E-07
mir-155	microRNA	-2.987	4.66E-04
RNF31	enzyme	-3	4.85E-04
NRAS	enzyme	-3.048	1.38E-13
GFI1	transcription regulator	-3.064	1.51E-04
miR-34a-5p (and other miRNA	mature microRNA	-3.148	6.46E-03
CBX5	transcription regulator	-3.162	2.75E-03
miR-155-5p (miRNAs w/seed	mature microRNA	-3.238	8.95E-03
PTGER4	G-protein coupled receptor	-3.256	4.51E-15
NCSTN	peptidase	-3.302	1.08E-08
RC3H1	enzyme	-3.302	2.96E-08
IL1RN	cytokine	-3.324	7.89E-09
APOE	transporter	-3.618	4.36E-16
MAPK1	kinase	-3.656	1.29E-10
ZC3H12C	other	-3.675	2.65E-13
SMARCA5	transcription regulator	-3.691	3.00E-13
PSMB11	peptidase	-3.71	5.10E-11
TGFBR2	kinase	-3.825	6.24E-08
PIK3CG	kinase	-4.055	2.36E-24
ACKR2	G-protein coupled receptor	-4.085	3.32E-15
PNPT1	enzyme	-4.333	1.32E-16
IL10RA	transmembrane receptor	-4.397	6.25E-47
SIRT1	transcription regulator	-4.401	1.40E-21
ETV6-RUNX1	fusion gene/product	-4.428	1.06E-11
HOXA10	transcription regulator	-4.447	1.25E-08
TRIM24	transcription regulator	-4.572	1.27E-15
INSIG1	other	-4.929	1.26E-16
SOCS1	other	-4.949	2.34E-21
Irgm1	other	-4.995	2.06E-18
mir-21	microRNA	-5.972	2.87E-19

C **27pepL**

UR: top 26

27pepL UR	Molecule Type	Activation z-score	B-H corrected p-value
TNF	cytokine	3.755	5.50E-10
CSF1	cytokine	2.935	1.38E-03
CD40	transmembrane receptor	2.716	5.53E-02
SIRT3	enzyme	2.629	5.13E-07
Interferon alpha	group	2.551	7.57E-03
Immunoglobulin	complex	2.533	6.94E-06
IL18	cytokine	2.531	6.77E-02
Tnf (family)	group	2.407	7.20E-02
IL3	cytokine	2.395	9.81E-03
mir-33	microRNA	2.236	2.78E-04
CCN5	growth factor	2.236	7.99E-03
IFNA1/IFNA13	cytokine	2.236	1.05E-02
miR-2392	mature microRNA	2.235	1.19E-06
FLCN	other	2.232	1.55E-03
mir-2392	microRNA	2.228	1.19E-06
N-cor	group	2.219	2.72E-04
TNFRSF9	transmembrane receptor	2.219	5.77E-04
CD28	transmembrane receptor	2.137	5.47E-02
IL15	cytokine	2.117	8.44E-02
CSF2	cytokine	2.079	7.02E-02
RELA	transcription regulator	2.052	3.97E-02
MEX3A	other	2	1.39E-02
MALAT1	other	2	1.76E-02
ASXL1	transcription regulator	2	1.87E-02
USP22	peptidase	2	1.98E-02
Map3k7	kinase	2	5.88E-02

UR: bot 30

MLXIPL	transcription regulator	-2.415	3.02E-03
BCL6	transcription regulator	-2.415	6.96E-02
MRPL12	other	-2.423	1.43E-08
HNF4Œ± dimer	complex	-2.425	1.33E-06
T3-TR-RXR	complex	-2.425	4.01E-04
PPARGC1B	transcription regulator	-2.438	5.49E-03
GC-GCR dimer	complex	-2.449	5.25E-06
LIF	cytokine	-2.559	6.36E-07
CREBBP	transcription regulator	-2.577	2.24E-03
SOCS1	other	-2.607	2.36E-02
CEBPA	transcription regulator	-2.615	2.05E-12
ESR2	ligand-dep. nuclear receptor	-2.643	7.74E-03
KDM6A	enzyme	-2.646	3.33E-05
PKD1	ion channel	-2.714	2.11E-03
NFE2L2	transcription regulator	-2.791	9.48E-09
ACOX1	enzyme	-2.794	2.61E-14
PPARGC1A	transcription regulator	-2.811	1.06E-05
Hmgn3	other	-2.813	6.93E-16
TWNK	enzyme	-2.813	3.82E-08
DAP3	other	-2.828	1.95E-10
LONP1	peptidase	-2.828	2.52E-04
FST	other	-2.985	1.22E-05
PEBP1	other	-3	3.08E-07
FASN	enzyme	-3.36	4.62E-12
NR3C1	ligand-dep. nuclear receptor	-3.428	1.51E-09
PPARG	ligand-dep. nuclear receptor	-3.498	5.96E-14
ADIPOQ	other	-3.797	7.23E-23
SMARCB1	transcription regulator	-4.078	4.41E-08
HNF4A	transcription regulator	-4.225	4.23E-07
HNF1A	transcription regulator	-4.457	2.73E-18

Figure S2. Upstream Regulators (UR) from IPA analyses, single therapies. (**A**) UR predicted in IL-18-treated tumors; (**B**) UR in IL27-treated tumors; (**C**) UR in IL-27pepL-treated tumors. *Red,* predicted as activated upstream regulators and ranked by z-score; *Green*, predicted as inhibited upstream regulators, ranked by z-score. Shown are the UR meeting the cutoff of activation z-score >2.0 or <-2.0 and a B-H corrected p value < 0.05.

A

27→18

UR: top 30

27_18 UR	Molecule Type	Activation z-score	B-H corrected p-value
IFNG	cytokine	3.681	1.04E-08
CSF1	cytokine	3.655	2.31E-06
IL21	cytokine	3.383	5.64E-04
TLR4	transmembrane receptor	3.204	1.40E-04
LDL	complex	3.177	5.42E-03
MYD88	other	3.06	8.44E-05
P38 MAPK	group	3.05	1.00E-02
TLR3	transmembrane receptor	2.92	4.62E-02
CSF2	cytokine	2.886	8.97E-03
IL2	cytokine	2.873	3.60E-02
IL5	cytokine	2.872	1.84E-02
PARP1	enzyme	2.809	7.00E-03
IL1B	cytokine	2.803	2.36E-07
FFAR3	G-protein coupled receptor	2.739	3.68E-06
TGM2	enzyme	2.714	1.02E-02
TNF	cytokine	2.681	1.50E-05
F2RL1	G-protein coupled receptor	2.621	1.37E-03
CARD9	other	2.611	1.86E-05
STAT1	transcription regulator	2.61	4.56E-11
APP	other	2.513	1.19E-06
IL17A	cytokine	2.482	1.20E-02
TNFSF11	cytokine	2.465	4.85E-03
USP22	peptidase	2.449	1.08E-03
SELPLG	other	2.449	1.08E-03
HRG	other	2.449	2.02E-05
LGALS3	other	2.425	5.16E-03
PF4	cytokine	2.416	1.19E-02
Ige	complex	2.412	1.54E-03
HMGB1	transcription regulator	2.41	5.40E-03
IL3	cytokine	2.4	1.96E-03

UR: bot 30

USF2	transcription regulator	-2.2	8.78E-03
AIRE	transcription regulator	-2.2	4.30E-03
RNF31	enzyme	-2.2	2.63E-03
Tcf 1/3/4	group	-2.2	4.15E-07
ST8SIA1	enzyme	-2.207	3.79E-04
B4GALNT1	enzyme	-2.207	4.17E-05
TRIM24	transcription regulator	-2.236	4.61E-02
HBB	transporter	-2.236	7.79E-03
LIPE	enzyme	-2.236	2.67E-05
PPARG	ligand-dependent nuclear re	-2.306	6.11E-08
PKD1	ion channel	-2.331	1.73E-03
SCD	enzyme	-2.387	1.96E-03
PXR ligand-PXR-Retinoic a	complex	-2.395	1.08E-03
BCL6	transcription regulator	-2.399	2.98E-02
mir-21	microRNA	-2.413	9.97E-05
HNF4Œ± dimer	complex	-2.425	3.70E-06
FST	other	-2.449	7.47E-03
SMTNL1	other	-2.449	1.50E-04
NR3C1	ligand-dependent nuclear re	-2.485	6.13E-05
ZC3H12C	other	-2.611	2.25E-05
KDM6A	enzyme	-2.646	8.99E-05
Hmgn3	other	-2.813	1.90E-13
CFTR	ion channel	-2.905	1.11E-06
PTGER4	G-protein coupled receptor	-2.97	2.87E-03
SOCS1	other	-2.985	1.48E-03
ACOX1	enzyme	-3.186	3.72E-13
IL10RA	transmembrane receptor	-3.595	6.65E-13
HNF4A	transcription regulator	-3.841	2.13E-09
ADIPOQ	other	-4.069	6.57E-20
HNF1A	transcription regulator	-4.345	2.81E-16

B

27pepL→18

UR: top 30

27pL 18 UR	Molecule Type	Activation z-score	B-H corrected p-value
CSF1	cytokine	3.773	9.94E-09
TNF	cytokine	3.481	1.23E-15
RELA	transcription regulator	3.326	1.63E-03
CSF2	cytokine	3.266	2.35E-03
Ige	complex	3.162	2.71E-05
TNFSF12	cytokine	3.062	1.12E-04
IL1B	cytokine	2.936	1.13E-09
TLR3	transmembrane receptor	2.889	2.27E-03
ERK1/2	group	2.883	1.12E-04
APP	other	2.866	1.93E-05
SPP1	cytokine	2.8	1.82E-03
MAPKAPK2	kinase	2.789	5.91E-05
IL33	cytokine	2.788	5.54E-06
KITLG	growth factor	2.778	6.71E-04
NFKB1	transcription regulator	2.723	3.44E-07
LDL	complex	2.672	1.15E-03
TNFSF11	cytokine	2.647	1.70E-06
IL1A	cytokine	2.647	2.54E-06
SIRT3	enzyme	2.629	1.02E-06
EGR1	transcription regulator	2.626	4.13E-05
CCN1	other	2.618	3.12E-03
cytokine	group	2.611	1.54E-02
SLC27A2	transporter	2.606	7.60E-05
IL18	cytokine	2.592	1.94E-03
Ccl2	cytokine	2.579	6.71E-05
IL21	cytokine	2.555	3.55E-03
MYD88	other	2.545	1.42E-04
MAP2K1	kinase	2.543	4.84E-05
HRG	other	2.449	1.15E-05
collagenase	group	2.449	1.44E-04

UR: bot 30

ALKBH1	enzyme	-2.236	1.71E-06
NSUN3	enzyme	-2.236	1.71E-06
PSEN1	peptidase	-2.277	8.86E-03
PXR ligand-R	complex	-2.395	6.54E-04
MLXIPL	transcription regulator	-2.415	1.21E-03
HNF4Œ± dim	complex	-2.425	2.11E-06
COP1	enzyme	-2.425	1.03E-04
PPARGC1B	transcription regulator	-2.438	6.08E-03
LONP1	peptidase	-2.449	1.89E-03
NR3C1	ligand-dependent nuclear	-2.531	4.72E-07
LIF	cytokine	-2.559	4.08E-07
MED1	transcription regulator	-2.563	1.20E-04
VIP	other	-2.617	9.43E-03
GFI1	transcription regulator	-2.63	4.58E-03
DAP3	other	-2.646	2.04E-08
KDM6A	enzyme	-2.646	4.98E-05
ADIPOQ	other	-2.721	1.45E-26
CFTR	ion channel	-2.744	5.92E-07
ADAM12	peptidase	-2.779	8.42E-07
Hmgn3	other	-2.813	6.32E-14
LIPE	enzyme	-2.813	7.47E-05
PPARGC1A	transcription regulator	-2.96	7.72E-07
BCL6	transcription regulator	-2.97	4.45E-02
FST	other	-2.985	2.06E-05
FASN	enzyme	-3.06	3.19E-09
SOCS1	other	-3.081	1.96E-04
SMARCB1	transcription regulator	-3.395	2.35E-08
PPARG	ligand-dependent nuclear	-3.483	3.05E-14
HNF4A	transcription regulator	-3.819	5.12E-10
HNF1A	transcription regulator	-4.457	1.32E-18

Figure S3. Upstream Regulators from IPA analyses for the combination therapies. Red, predicted as activated upstream regulators and ranked by z-score; Green, predicted as inhibited upstream regulators, ranked by z-score. Shown is also the p-value of the overlap.

Supplementary Table

Table S1. Gene expression signatures utilized in the EPIC RNAseq cell profiling analyses.

Signature name	Bulk Expression File	FKPM, Counts	Source	Reference Expression File	Source
LMPP_gsea	GSE116177	FKPM	Haemopedia	GSE15330, M7021	*GSEA, immusigdb*
GMP_gsea	GSE116177	FKPM	Haemopedia	GSE15330, M7041	*GSEA, immusigdb*
MPP_gsea	GSE116177	FKPM	Haemopedia	GSE22432,M7817	*GSEA, immusigdb*
gMDSC_gsea	GSE140029	FKPM	Ref. [42]	GSE24102, M4541	*GSEA, immusigdb*
mMDSC_gsea	GSE140029	FKPM	Ref. [42]	GSE23502, M8087	*GSEA, immusigdb*
MDSC_gsea	GSE140029	FKPM	Ref. [42]	GSE21927, M7563	*GSEA, immusigdb*
HSC1_gsea	GSE109125	Cts	ImmGen ULI	GSE15330, M7060	*GSEA, immusigdb*
HSC2_gsea	GSE109125	Cts	ImmGen ULI	GSE15330, M7018	*GSEA, immusigdb*
Macro_gsea	GSE109125	Cts	Immgen	GSE27859, M8590	*GSEA, immusigdb*
NKT_gsea2	GSE109125	Cts	Immgen	GSE27786, M4870	*GSEA, immusigdb*
CD8_Teff_gsea	GSE109125	Cts	Immgen	GSE1000002, M3027	*GSEA, immusigdb*
Bcell_gsea	GSE109125	Cts	Immgen	GSE27786, M4799	*GSEA, immusigdb*
Neutr_gsea	GSE109125	Cts	Immgen	GSE27786, M4880	*GSEA, immusigdb*
Treg_gsea	GSE109125	Cts	Immgen	GSE13306, M3229	*GSEA, immusigdb*
CD8_T_gsea	GSE109125	Cts	Immgen	GSE1000002, M3035	*GSEA, immusigdb*
cDC_gsea	GSE109125	Cts	Immgen	GSE3691, M6363	*GSEA, immusigdb*
DC8a_gsea	GSE109125	Cts	Immgen	GSE12392 M7135	*GSEA, immusigdb*
pDC1_gsea	GSE109125	Cts	Immgen	GSE22432, M7835	*GSEA, immusigdb*
pDC2_gsea	GSE109125	Cts	Immgen	GSE3691, M6368	*GSEA, immusigdb*
NK11_gsea	GSE109125	Cts	Immgen	GSE13229, M3213	*GSEA, immusigdb*
NK27_gsea	GSE109125	Cts	Immgen	GSE13229, M3205	*GSEA, immusigdb*
NK27_11_gsea	GSE109125	Cts	Immgen	GSE13229, M3209	*GSEA, immusigdb*
M2_gsea	GSE109125	Cts	Immgen	GSE18804, GEO2R analysis	*GSEA, immusigdb*
Tgd_gsea	GSE109125	Cts	Immgen	GSE22196, M7655	*GSEA, immusigdb*
Th2_gsea	GSE109125	Cts	Immgen	GSE14026, M3164	*GSEA, immusigdb*
Th1_gsea	GSE109125	Cts	Immgen	GSE14026, M3356	*GSEA, immusigdb*
NKT_gsea	GSE109125	Cts	Immgen	GSE3039, M6429	*GSEA, immusigdb*
M1_gsea	GSE109125	Cts	Immgen	GSE26912, M8205	*GSEA, immusigdb*
Plasma_gsea	GSE109125	Cts	Immgen	GSE4142, M6395	*GSEA, immusigdb*

Shown are signature names, bulk and reference files used and sources.
